# Frequency of rpoB, katG, and inhA Gene Polymorphisms Associated with Multidrug-Resistant *Mycobacterium tuberculosis* Complex Isolates among Ethiopian TB Patients: A Systematic Review

**DOI:** 10.1155/2022/1967675

**Published:** 2022-06-16

**Authors:** Aynias Seid, Nega Berhane, Semira Nureddin

**Affiliations:** ^1^Department of Medical Biotechnology, Institute of Biotechnology, University of Gondar, Gondar, Ethiopia; ^2^Department of Biology, Faculty of Natural and Computational Science, Debre-Tabor University, Debre-Tabor, Ethiopia; ^3^Department of Biology, Faculty of Natural and Computational Science, Woldia University, Woldia, Ethiopia

## Abstract

Tuberculosis (TB) is one of the top 10 causes of mortality and the first killer among infectious diseases of poverty (IDoPs) worldwide. It disproportionately affects on-third of the world's low-income countries including Ethiopia. One of the factors driving the TB epidemic is the global rise of MDR/XDR-TB and their low detection affect the global TB control progress. Recently, the resistance-associated genetic mutations in MTBC known to confer drug resistance have been detected by rapid molecular diagnostic tests and sequencing methods. In this article, the published literature searched by PubMed database from 2010 to 2021 and English language were considered. The aim of this systematic review was to assess the prevalence of the most common rpoB, katG, and inhA gene mutations associated with multidrug resistance in MTBC clinical strains among TB patients in Ethiopia. Though 22 studies met our eligibility criteria, only 6 studies were included in the final analysis. Using the molecular GenoType MTBDRplus and MTBDRsl line probe assay and sequencing procedures, a total of 932 culture-positive MTBC isolates were examined to determine RIF, INH, and MDR-TB resistance patterns along with rpoB, katG, and inhA gene mutation analysis. As a result, among the genotypically tested MTBC isolates, 119 (12.77%), 83 (8.91%), and 73 (7.32%) isolates were INH, RIF, and MDR-TB resistant, respectively. In any RIF-resistant MTBC strains, the most common single point mutations were in codon 531 (S531L) followed by codon 526 (H526Y) of the rpoB gene. Besides, the most common mutations in any INH-resistant MTBC were strains observed at codon 315 (S315T) and WT probe in the katG gene and at codon C15T and WT1 probe in the inhA promoter region. Detection of resistance allele in rpoB, KatG, and inhA genes for RIF and INH could serve as a marker for MDR-TB strains. Tracking the most common S531L, S315T, and C15T mutations in rpoB, katG, and inhA genes among RIF- and INH-resistant isolates would be valuable in TB diagnostics and treatment regimens, and could reduce the development and risk of MDR/XDR-TB drug-resistance patterns.

## 1. Introduction

Currently, tuberculosis (TB) is the first leading fatal chronic infectious disease worldwide, which is caused by the *Mycobacterium tuberculosis* complex (MTBC). TB remains a major global public health problem to date, predominant in middle- and low-income countries including Ethiopia. TB is one of the top 10 leading causes of death and the top killer among infectious diseases [1]. It is estimated that one-third of the world's population is latently infected with TB [2].

According to the World Health Organization (WHO) Global Tuberculosis Report 2021, there were an estimated 9.9 million people infected with new TB cases and 1.3 million deaths in 2020. A total of 400,000 of new TB cases were rifampicin resistant (RIFR-TB) and 68% of them had multidrug resistance tuberculosis (MDR-TB) (defined as resistant to the two most effective first-line drugs isoniazid (INH) and rifampicin (RIF) for the treatment of TB patients). Most TB cases were in the WHO regions of South-East Asia (43%) and Africa region (25%) [3]. Ethiopia is one of the 22 high burden countries (HBC) in all three lists, TB, HIV-associated TB, and MDR-TB in the world, which are becoming pressing challenges in the efforts to control TB in Ethiopia. The WHO annual TB 2021 reported that there were an estimated 108,000 TB cases, of which 13.6% were MDR-TB (1.61% among new- and 12% pre-treated TB cases) with 4 in 100 people dead, and ranked 12th in the world and 4th in Africa region in 2020 [3].

Effective management of TB relies on a prompt diagnosis, rapid detection of drug resistance, and fast initiation of an effective treatment regimen [4]. Antimicrobial drug combination therapy is one of the effective strategies used to control TB. However, to date, the challenging condition for the global prevention and control program of TB and the major factors fueling the TB epidemic is the emergence and spread of multidrug- and extensively drug-resistance tuberculosis (MDR/XDR-TB) strains of MTBC on new and previously treated cases [5]. Early case detection and treatment of MDR/XDR-TB cases is essential to prevent and control the transmission of TB [6], and has become an urgent public health problem in developing countries including Ethiopia, due to their complex diagnostic and treatment obstacles [7]. Some of the significant factors associated with increasing the development of drug-resistant tuberculosis (DR-TB) and the risk of direct transmission of DR-TB are an increase of TB with HIV-1 co-infection, overcrowded living conditions, lack of or poor access to healthcare such as lack of DR-TB diagnostic tools and delaying drug susceptibility testing (DST) practices, inadequate administration of anti-TB therapy regimens with inappropriate prescription of anti-TB drugs and patient compliance [8], weak TB prevention and control program, and high prevalence of diabetes mellitus, alcoholism, and smoking [9, 10]. It has been recognized as a poverty-related disease.

Unlike other pathogenic bacteria, resistance to anti-TB drugs in MTBC arises as a result of spontaneous chromosomal mutations in a specific gene called single nucleotide polymorphisms (SNPs), that reduce the bacterium's susceptibility to the antimicrobial agents and influence the efficacy of anti-TB treatments, because tubercle bacilli have no known efficient mechanism for horizontal gene transfer [11]. This knowledge has been exploited in the development of molecular diagnostic tools as a rapid alternative to conventional culture-based drug-susceptibility testing [12]. The WHO has endorsed the commercially available molecular Line Probe Assays (LPAs), the GenoType MTBDRplus assay (Hain Lifescience, Nehren, Germany) to detect the presence of MTBC together with the most common rpoB, inhA, and katG genetic mutations that confer resistance to the most common first-line anti-TB drugs of RIF and INH [13]. Multiple reviews have identified genes that encode drug targets and have summarized the various mechanisms of resistance to both INH and RIF [14, 15]. Moreover, greater than 95% of RIF resistance is associated with mutations in an 81 base pair section of the rpoB gene, while INH resistance appears more complex and has been associated with multiple genes, most frequently katG gene and inhA promoter region [8, 16–18].

RIF is one of the most potent first-line anti-TB drugs and has very effective bactericidal activity against MTBC during TB treatment. Resistance to RIF is the most important indicator of MDR-TB and serves as a surrogate marker for the detection of MDR-TB [8]. The primary mechanism of RIF resistance is due to the mutations in the rpoB gene that encode the DNA-dependent RNA polymerase *β*-subunit [19, 20]. RIF resistance is largely associated with the most common mutations of the rpoB gene within 81-bp fragment of RIF resistance determining region (RRDR) or hot-spot region at codon rpoB 531, rpoB 526, and rpoB 516 between codons 507 and 533 [17, 18, 21]. INH resistance appears more complex and has been associated with multiple genes, most commonly katG gene that codes for a catalase peroxidase and the promoter region of the inhA gene. INH resistance rely on detection of the mutations at codon 315 (katG315) and position –15 (inhA-15) promoter region [9, 12].

So, it is significant to explore the prevalence of resistance-conferring genetic mutations in MTBC in more detail for understanding the drug-resistance mechanism. Then, the objective of this study was to assess the prevalence of rpoB, katG, and inhA gene polymorphisms associated with MDR-TB in Ethiopia based on previously published original research articles data.

## 2. Methods

### 2.1. Literature Search Strategy

Clinical peer-reviewed publications written only in English language were searched on PubMed database as well as Google Scholar for assessing the rpoB, katG, and inhA gene mutations associated with RIF- and INH-resistant MTBC strains. The search for published studies was limited from January 2010 to December 2021 year of publication. We used the following database key search words individually as well as in combination applying the “AND” operator: “rifampicin,” “isoniazid,” “RIF,” “INH,” “Mycobacterium tuberculosis complex,” “MTBC,” “tuberculosis,” “rpoB,” “katG,” “inhA,” “drug-resistance,” “multidrug-resistance,” “drug susceptibility testing,” “molecular diagnostics,” “Line Probe Assay,” “GenoType®MTBDR plus assay,” “GenoType®MTBDR sl assay,” “molecular characterization,” “molecular detection,” “DNA sequencing,” “gene mutations,” and “Ethiopia” in various combinations.

### 2.2. Screening and Eligibility of Studies

The published article screening for this review was done in three stages: looking at the title, abstracts, and then full-text article eligibility. Furthermore, we reviewed the potential studies that reported gene mutations associated with RIF and INH drug resistance in MTBC strains in Ethiopia were included in the analysis. Articles retrieved from the online databases were imported into the Mendeley reference software. Duplicate published articles of the same study were removed from Mendeley software and excluded from the analysis.

### 2.3. Study Selection Inclusion and Exclusion Criteria

All clinical studies (cross-sectional, case–control, and cohort studies) that met the following inclusion criteria were selected and included: studies were written in English language; presented original data; studies used clinical strains of MTBC; studies that reported data regarding the mechanisms of anti-TB drug resistance or rpoB, katG, and inhA gene mutations associated with anti-TB drug resistance or MDR-TB in clinical MTBC strains among pulmonary TB (PTB) and extrapulmonary TB (EPTB) patients (both retreated and newly diagnosed cases); studies that performed liquid- and/or solid-based culture media; studies that used WHO-approved commercial or in-house molecular diagnostics tools such as LPAs and DNA sequencing as a means of characterizing mutations; and TB research conducted in Ethiopia. We excluded the published studies from the analysis with the following exclusion criteria: studies that did not report mechanisms of anti-TB drug resistance or gene mutations associated with RIF and INH resistance or MDR-TB in clinical MTBC strains; studies reporting data on non-tuberculous mycobacteria; studies that did not perform culturing and phenotypic DST tool to first-line RIF and INH drugs; studies that did not have complete reference standard; and Editorial reports, case reports and review articles were excluded from the analysis.

### 2.4. Data Acquisition

Information or data from each published article were recorded appropriately. The two investigators (AS and SN) independently collected the relevant information correlated with the study characteristics as follows: primary author name, journal name, year of publication, study area (geographic origin of specimens), study population, sample size, year(s) of sample collection (study period), study design, patient age range, type of TB patient cases, cell culturing method (Table 1), diagnostic method (phenotypic DST and genotypic testing method), total positive cases with culture, total number of resistant and susceptible MTBC isolates, frequency of anti-TB drug resistance (any INH or RIF resistance, and MDR-TB), location of gene mutation among individual isolate, amino acid and specific nucleotide (codon changes in each resistant gene), and frequency of mutations in the rpoB, katG, and inhA gene associated with RIF and INH drug resistance (Table 2). All the relevant data were compiled using MS Excel software (Microsoft, Redmond, WA).

### 2.5. Quality Assessment of Articles

The critical quality of each full-text article was evaluated by investigators (AS and SN) independently using the following article quality assessment checklists: details of study subjects and the study settings, appropriate statistical analysis, reference standard, and publication quality. Finally, investigator differences were resolved through discussion to reach a consensus and to include the published articles in the final analysis. Each article with a “YES” score of four and above was considered good quality and was included in the final analysis, while studies with an average “YES” score of below three were considered poor quality and were excluded from this systematic review analysis.

### 2.6. Data Processing and Statistical Analysis

Relevant data were extracted from the included articles using a standard format prepared in Microsoft Excel. All the collected statistical data were further entered and analysed with the Statistical Package for the Social Sciences (SPSS) version 23.0 software (IBM SPSS, Chicago, IL, United States). The frequency of any anti-TB drug resistance and resistance to INH and RIF were extracted from each included study. Each article was examined for individual mutations and a combination of mutations in rpoB, katG, and inhA genes. Mutations in the same locus but with a different change were reported independently. The frequency of each resistant gene distinct mutation or cumulative/combinations of mutations (mutations harbored by the same isolate) in resistant isolates was calculated as the number of resistant MTBC isolates for a particular or combination of anti-TB drug in which the mutation was found, divided by the total number of resistant isolates tested across all included studies. Similarly, the rate of each nucleotide (codon) changes at each resistant gene locus/probe (rpoB, katG, and inhA) was calculated out of the total resistant genes.

## 3. Results

### 3.1. Study Search Results

**Figure 1:**
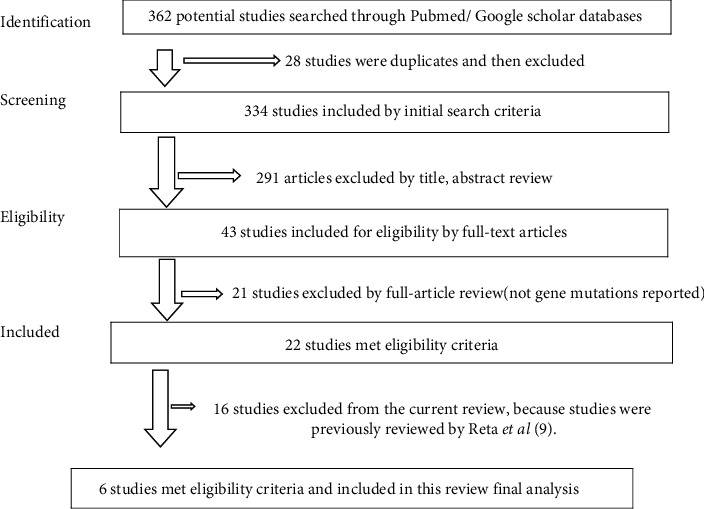
Flow diagram illustrating literature search strategy.

As revealed in Figure 1, (a) total of 362 potential studies published between 2010 and 2021 were searched from the electronic database sources using initial search parameters. Of the total articles, 28 published articles were duplicated and then excluded from further evaluation as they did not meet inclusion criteria, 291 studies were excluded based on their title and abstract and they did not meet inclusion criteria, while 43 published articles were subjected to full-text article review. After full-text evaluation, 22 studies on the impact of rpoB, katG, and inhA gene mutations associated with MDR-TB drug-resistance patterns in Ethiopia met all eligibility criteria, however, 16 studies were excluded because they were previously reviewed, and finally only 6 articles were included in this systematic review final analysis [22–27].

### 3.2. Individual Study Quality Assessment Results

Based on the article quality assessment checklists (details of study subjects and the study settings, appropriate statistical analysis, reference standard, and publication quality) the quality of each individual study was assessed twice per two investigators (AS and SN). Each of the six published articles was scored four and more “YES” per article quality assessment criteria and were considered as high quality based on the above paper quality assessment criteria and were included in this systematic review final analysis.

### 3.3. Description of Studies Included in the Systematic Review

As shown in Table 1, a total of 22 studies met the eligibility criteria, but 16 studies were reviewed previously by Reta et al. [9] and excluded from the current systematic review, and only 6 studies with 1711 TB study participants were included and evaluated in this systematic review final analysis [22–27]. According to the year of publication, of the six included studies, five (83.33%) articles got published in 2021 and the remaining one (16.67%) was published in 2020. Whenever, the geographical origin of MTBC strains in these studies is considered as two studies each were from Amhara region [22, 26] and Tigray region [25, 27], one study from Addis Ababa [24], and the other one study was performed in multiple regions of Oromia, Amhara, and South Nation Nationality and their People [23]. Based on study design, five studies used a cross-sectional type of study design [22, 24–27] and one study did not report the study design [23]. A total of four studies with 1236 clinical isolates were collected from pulmonary tuberculosis (PTB) [22, 24, 25, 27], one study performed from both PTB and EPTB [26], and one study from tuberculosis lymph node (TBLN) patients [23]. Among all studies, two studies were from new TB cases [25, 27], three studies of the participant were from both new and pre-treated TB cases [22, 23, 26], and one study did not report the study participant information [24].

### 3.4. Prevalence of Multidrug-Resistant MTBC

As described in Table 2 of this review, out of the six included studies, only two studies with 195 MTBC isolates were identified by conventional phenotypic DST and reported the prevalence of MDR-TB strains on a Lowenstein–Jensen [14, 18], of which 137 (70.26%) MTBC isolates were susceptible for all first-line drugs, 54 (27.69%) isolates were any drug resistance, 32 (16.41%) were INH resistant, 18 (9.23%) were RIF resistant, and 11 (5.64%) were MDR-TB.

In this study, all the included studies used the molecular diagnostic tools for the detection of drug-resistance MTBC strains, GenoType® MTBDRplus assay was the most common molecular DST method used [22–24,26], one study used GenoType MTBDRplus and MTBDRsl LPAs [25], and the other study done by whole genome sequencing (WGS) [27]. As a result, based on the above molecular diagnostic methods, all included studies, except one, reported the prevalence of MDR-TB strains [23–27]. Overall, 932 culture-positive MTBC isolates were tested to identify RIF, INH, and MDR-TB resistance patterns and the rpoB, katG, and inhA gene mutation analysis using genotyping resistance tests. The prevalence of any INH resistance was 119 (12.77%) and any RIF resistance was 83 (8.91%). Besides, the prevalence of MDR-TB was 73 (7.32%).

### 3.5. Frequency of rpoB, katG, and inhA Genes Mutations

As shown in Table 2, five studies reported the frequency of mutations and nucleotide (codon) changes in the rpoB, katG genes, and inhA region [22–26], while one study reported the rpoB and katG genes mutations among MDR-TB distinct isolates [27]. Mutation data (mutation location, original amino acid and nucleotide, and mutated amino acid and nucleotide) and frequencies in the rpoB, katG, and inhA gene are presented in Table 2.

A total of 83 MTBC strains with any RIF resistance were identified by standard WHO-approved molecular diagnostic tools, the most commonly occurring SNPs are in RIF-resistant isolates, at position 531 of the rpoB gene. In this article, the most common mutations of the rpoB gene found in the rpoB S531L (34.01%), followed by the rpoB S450L (19.78%), rpoB WT8 probe (15.38%), rpoB WT7 probe (4.4%), and rpoB H526Y (4.4%) among RIF-resistant MTBC strains. Also, the most common mutations of the katG gene in any INH-resistant MTBC strains were observed in the katG S315T (68.6%) and katG WT probe (12.4%). In the inhA promoter region, the most frequent mutations were observed in the inhA C15T (11.57%), inhA WT1 probe (4.13%), inhA WT2 probe (0.83%), and inhA MUT1 probe (0.83%). Moreover, combination mutations were observed in the WT1+MUT1 (1.65%) in both the katG gene and the inhA promoter region.

## 4. Discussion

Recently, the global TB control and prevention program is challenging due to the emergence and spread of MDR/XDR-TB. This drug resistance in MTBC isolate is associated with chromosomal genes mutations (e.g., rpoB, katG, inhA, pncA, embB, rrs, gyrA, and gyrB) [14, 28], rather than by horizontal gene transfers (HGT) include plasmids or transposons [29]. Rapid diagnosis and accurate detection of chromosomal gene mutations in resistance-determining regions in all forms of DR-TB is a key factor for effective patient care and for reducing the spread of these resistant strains. A periodic assessment of the frequency of gene mutation in drug-resistance tuberculosis in high TB burden countries is essential to identify early and address the challenges of DR-TB transmission. This helps to enhance the TB prevention and control program performance and achieve the end TB strategy goals.

Globally, the use of molecular-based diagnostic methods such as GenoType MTBDRplus and MTBDRsl LPAs and WGS are more efficient and effective tools for the detection of specific gene mutations associated with anti-TB drug resistance by reducing the turnaround time required to diagnose cases from weeks to hours. This review included molecularly diagnosed 932 culture-positive MTBC isolates obtained from newly diagnosed TB patients. From the tested isolates, 119 (12.77%), 83 (8.91%), and 73 (7.32%) isolates were INH, RIF, and MDR-TB resistant, respectively.

In the case of RIF-resistant MTBC isolates, this review found that the most gene mutation was in codons 531 (34.01%), 526 (9.3%), and 516 (2.33%) in RIF resistance determining region (RRDR) of the rpoB gene, whereas the most associated mutations with RIF resistance was in codon 531 followed by 526 mutations. There was a similar report in the previous systemic review that indicated the genetic variation to be single base substitutions with the most common mutation in the rpoB gene encoding the *β*-subunit of DNA-dependent RNA polymerase was observed at codon 531 (S531L) [8,9]. Similarly, a study conducted in Kyrgyz Republic explained that up to 95%–98% of RIF-resistant strains exhibit mutations in rpoB gene at 531 codon [30], while in Vietnam and Sri Lanka about 30%–48% of RIF-resistant strains exhibit mutations [31, 32]. In line with previous and our findings, globally the association between rpoB gene and RIF-resistant MTBC isolates is wide with S531L mutation. Moreover, in this study, the most common gene mutations responsible for INH resistance in MTBC isolates were found in codon 315 (S315T) (68.6%) in the katG gene and C15T (11.59%) nucleotide changes in the inhA promoter region. The frequency patterns of the most common mutations associated with INH resistance appear to differ between individual genes. It is clear that the overwhelming majority of 315 mutations 64% in the katG gene and inhA 15 mutation is the dominant (19%) mutation in the inhA promoter region [33]. This analysis supports the association between the resistance-conferring mutations due to the katG gene at the 315-codon position (S315T) and high-level INH resistance in MTBC isolates, that increase the development of MDR-TB. In the same point of view, previously in different TB epidemic countries including Vietnam, Kyrgyz Republic, and Ethiopia, the frequency of resistant-conferring mutations at rpoB, katG, and inhA in MDR-TB isolates was investigated. The most common point mutations were at codons rpoB531 (37.8%), rpoB526 (23%), rpoB516 (9.46%), and nucleotide substitution at codon katG315 (76.83%) in Vietnam [31], while in Kyrgyz Republic, the common mutations were in codons rpoB531 (64.8%), rpoB526 (17.3%), rpoB 516 (8.1%), and at codon katG 315 Ser315⟶Thr (88.6%) [30]. Moreover, a similar study was done by Reta et al. in Ethiopia, the frequency of rpoB, katG, and inhA mutations were observed in the rpoB MUT3(S531L) probe (550 cases), rpoB WT8 probe (224 cases), rpoB WT7 probe (91 cases), rpoB MUT2A(H526Y) (68 cases), rpoB MUT2B(H526D) (40 cases) in RIF-resistant strains, and katG MUT1 probe (860 cases), katG WT probe (309 cases), inhA MUT1 probe (inhA C15T; 31 cases), and inhA WT1 probe (30 cases) in INH-resistant strains [9]. So, our findings have more similarities with the above previous results except the MTBC isolates used were high in number.

This is the second study to assess the prevalence of rpoB, katG, and inhA gene mutations associated with MDR-TB isolates in Ethiopia. There are some limitations in this study that could be addressed in future research. This review focused only on the systematic review of the resistance-conferring gene mutations in MTBC strains from newly diagnosed TB patients. We did not perform the meta-analysis. This review only considered the published articles in the English language and included them in the final analysis. Another limitation of the current systematic review was that it only included a small number of articles in the final analysis because most of the previous studies related to gene mutations in INH- and RIF-resistant TB were reviewed by Reta et al. [9]. Several studies have shown that mutations in the rpoB, katG, and inhA genes were different from one region of the world to another TB endemic region and depend on the time of sample collection and other external factors. This kind of scientific study on the most common resistance-conferring rpoB, katG, and inhA gene mutations have great significant value in early diagnostic and treatment aspects, and TB infection control program in this TB epidemic area. Nevertheless, in Ethiopia, there were very limited numbers of scientific studies (not more than 25 studies) on gene mutations in DR-TB strains. Therefore, further research on the detection of rpoB, katG, and inhA gene mutations in any forms of DR-TB strains is needed to describe with enough depth and clarify the behavior of the mutations in DR-TB isolates in Ethiopia.

## 5. Conclusion and Recommendation

An understanding of the mechanism of drug resistance in MTBC at the molecular level will enable us to develop novel and rapid molecular tests. It deserves further investigation to determine which gene mutations may play a critical role in the epidemic of MDR/XDR-TB isolates in geographic settings. The increased proportion of resistance to RIF, INH, and MDR-TB in patients among new and previously treated cases indicates a need for better patient management to help prevent the evolution of drug-resistance TB [22, 23, 26]. However, the increasing frequencies of rpoB, katG, and inhA gene mutations in MDR-TB appear to vary by geographical locations and tie of sample collection [7]. This would permit modifying molecular tests to specific geographical regions and better multidrug combinatory therapy recommendations. In Ethiopia, the replacement of Ser531⟶Leu in rpoB gene, and Ser315⟶Thr mutation in katG gene could likely be the commonest variant of RIF and INH resistance, respectively. MDR-TB strains in Ethiopia likely developed most of their resistance because of combined mutations Ser531⟶Leu in rpoB gene and Ser315⟶Thr in katG gene. Taken together, this analysis will help guide the treatment of TB patients with resistant strains and reduce the overall burden of the disease in the country.

## Figures and Tables

**Table 1:** Description of studies included in this systematic review.

1st author name	Publication year	Publisher name	Study area	Study period	Study design	Study population	Sample size (n)	TB patients' cases	Sex ratio	Patient age range	Cell culturing method	Total positive cases (n)
Yigzaw et al. [22]	2021	DovePress	Amhara region	2018–2019	Prospective cross-sectional study	PTB patient	376	New and relapse	53.7% male and 46.3% female	16–88	LJ and MGIT 960 media	176
Ayalew et al. [23]	2021	DovePress	Bishoftu, gondar, mekele and Hawass	2016–2017	NR	TBLN patients	91	New and re-treated cases	40.7% male and 59.3% female	9–76	NR	NR
Tilahun et al. [24]	2020	PLOS	Addis Ababa	2017	Cross-sectional study	PTB patient	260	NR	56.3% male and 43.7% female	>15	LJ medium	190
Welekidan et al. [25]	2021	ELSEVIER	Tigray region	2018–2019	Cross-sectional study	PTB patient	300	New	63.4% male and 36.6% female	>15	LJ and MGIT 960 media	227
Gashaw et al. [26]	2021	BMC	Oromia Special Zone and Dessie Town	2015–2017	Cross-sectional study	PTB and EPTB patient	384	New and pre-treated cases	55.5% male and 45.5% female	18–75	LJ medium	112
Welekidan et al. [27]	2021	Frontiers	Tigray region	2018–2019	Cross-sectional study	PTB patient	300	New	63.4% male and 36.6% female	>15	LJ and MGIT 960 culture	227
Total							1711					932

*Note.* PTB, pulmonary tuberculosis; EPTB, extra-pulmonary tuberculosis; TBLN, tuberculosis lymph node; NR, not reported.

**Table 2:** Diagnostic results and rpoB, katG, and inhA gene mutation with MDR-TB drug-resistance pattern in *Mycobacterium tuberculosis*

1st author name	Conventional phenotypic DST							Molecular diagnostic tool(s)	Genotypic DST (molecular assay)			rpoB, katG, inhA gene polymorphism
	Total isolates with DST test (n)	Undetected (n)	Susceptible for all drugs (n)	Any drug-resistance (n)	INHR (n)	RIFR (n)	MDR (n)		INHR (n)	RIFR (n)	MDR (n)	
Yigzaw et al. [22]	126	NR	83	43	25	12	6	GenoType® MTBDRplus v2.0	17	3	NR	rpoB WT2 (1), rpoB WT8 S450L (S431L) (1), rpoB WT8 L452P (L533P) (1) katG S315T1 (12) inhA C15T (7)
Ayalew et al. [23]	NS	NS	NS	NS	NS	NS	NS	GenoType MTBDRplus VER 2.0.	6	2	2	rpoB: S531L (2), katG: S315T1 (3), S315T2 (1) inhA: C15T (1), unknown-inhA MUT1 (1)
Tilahun et al. [24]	NS	NS	NS	NS	NS	NS	NS	GenoType MTBDRplus	13	2	2	rpoB: S531L (1), WT7/MUT2A (H526Y) (1) katG: S315T1 (10) inhA: C15T (4) missed, WT1+MUT1 (katG + inhA (2)
Welekidan et al. [25]	NS	NS	NS	NS	NS	NS	NS	GenoType MTBDRplus and MTBDRsl	41	40	38	rpoB: S531L (28), H526Y (4), H526D (3), D516V (2), unknown – poB WT3 (2), rpoB WT4 (1), rpoB WT6 (1), rpoB WT8 (2), rpoB WT7 (2), katG: S315T (32), unknown- katG WT (7) inhA: C15T (2), unknown- inhA WT2 (1)
Gashaw et al. [26]	69	4	54	11	7	6	5	GenoTypic MTBDRplus assay	16	14	8	rpoBWT8 missed (10), WT3 (1), WT4 (1), WT6 (1), WT7 (1) katGWT missed (8), inhAWT1 (-15/-16) (5)
Welekidan et al. [27]	NS	NS	NS	NS	NS	NS	NS	Whole-genome sequencing (WGS)	26	25	23	rpoB: S450L (18), H445Y (1), H445D (1), D435V (1), H445N (2), D435Y (1), L430P (1) katG: S315T (25)

*Note.* NS, not studied; NR, not reported.

## Data Availability

The data sets analyzed during this article are available from the corresponding author upon reasonable request.

## Abbreviations

DST: Drug susceptibility testing
EPTB: Extrapulmonary tuberculosis
HBC: High burden country
LPAs: Line probe assays
MDR-TB: Multi-drug resistance tuberculosis
MTBC: *Mycobacterium tuberculosis* complex
PTB: Pulmonary tuberculosis
RRDR: RIF-resistance determining region
SNPs: Single nucleotide polymorphisms
TB: Tuberculosis
WGS: Whole genome sequencing
WHO: World Health Organization
XDR-TB: Extensively drug-resistance tuberculosis
